# “Are they medically fit?” - clinical audit on the physical assessment of mental health patients in A&E

**DOI:** 10.1192/bjo.2021.231

**Published:** 2021-06-18

**Authors:** Ashley Cooper

**Affiliations:** Greater Manchester Mental Health Trust

## Abstract

**Aims:**

A&E departments are busy places; with quick triage decisions required to prioritise urgent care to those who need it. This requires the use of predictions based on past experiences and probabilities. However, this runs the risk of patients being categorised by the prejudices and stigmas associated with their conditions; particularly in the case of mental health patients and the assumption they are otherwise ‘medically fit’. This is especially of concern when considering that mental health often deteriorates during acute physical illness.

Following a number of dangerous ‘near misses’, this audit was conducted to review the practice of triage and physical assessment of patients presenting to A&E with mental health symptoms. The aim was to compare practice against the Royal College of Emergency Medicine (RCEM) guidelines, to identify repeated issues and systemic vulnerabilities which endangered patients through a lack of appropriate assessment.

**Method:**

Using the online Electronic Patient Record (EPR) system, the notes of 100 patients referred to the Bolton Mental Health Liaison Team (MHLT) from Bolton A&E were reviewed. They were assessed for whether or not the patients had been appropriately physically assessed, according to the RCEM guidelines, before being referred to the MHLT. These results were analysed anonymously.

**Result:**

The findings showed that less than half (44%) of all referred patients had physical observations taken at all, and even fewer (37%) received the full, physical assessment before referral. Out of the patients identified as having abnormal physical observations only 58% were acted on. Many patients had no history or triage assessment completed; with triage referrals consisting of only the words “mental health”. Most importantly, the audit identified this lack of adequate physical assessment resulted in a 2% ‘near miss’ rate, including a missed diabetic ketoacidosis and delayed treatment for a missed overdose.

**Conclusion:**

Following this audit and the above result, it is clear that triage and physical assessment of mental health patients attending A&E is inadequate; with resulting risk of severe consequences to patients. It is therefore recommended to co-develop joint guidelines and teaching to guide A&E and MHLT practitioners on the process of completing the physical assessment prior to referral. It is also recommended to repeat this audit throughout other hospital trusts, in order to review the local referral pathways to ensure adequate physical assessment to avoid any ‘near misses’ or serious incidents.

